# A descriptive study of demographics, triage allocations and patient outcomes at a private emergency centre in Pretoria

**DOI:** 10.4102/safp.v63i1.5308

**Published:** 2021-11-09

**Authors:** Kirsty Hedding, Enrico Dippenaar, Lee Wallis

**Affiliations:** 1 Division of Emergency Medicine, Faculty of Health Sciences, University of Cape Town, Cape Town, South Africa

**Keywords:** emergency medicine, triage, ICU, critically ill patients, private healthcare

## Abstract

**Background:**

Triage aims to detect critically ill patients and to prioritise those with time-sensitive needs, whilst contributing to the efficiency of an emergency centre (EC). International systems have been relatively well researched; however, little data exists on the use of the South African Triage Scale (SATS) in private healthcare settings in South Africa (SA).

**Methods:**

A retrospective descriptive study was undertaken. Data relating to demographics, application of triage, time in EC and disposition were collected on all patients presenting to the EC from 1st January to 31st December 2018.

**Results:**

A total of 29 055 patients’ data were included. The mean age was 41 years. Most patients were triaged yellow (73.5%); 17.4% were triaged as red and orange. Patients were seen by a doctor in a mean time of 28 min. Delays to be seen exceeded standards for red and orange patients at 8 min and 18 min, respectively. Most patients (76.1%) were discharged; 5.6% were admitted to intensive care unit (ICU)/high care, and 14.4% to general wards. Of patients triaged red and orange, 11.1% and 49.3% were discharged, respectively, whereas 81.7% of yellow patients were discharged home.

**Conclusion:**

This study found that most patients were triaged into low acuity categories and were discharged home. High acuity patients were usually admitted to ICU/high care; however, these patients experienced delays in receiving treatment. The causes of these issues, and the implications, remain unknown. Large numbers of high acuity patients were discharged home. Further studies are needed to understand the influence of triage accuracy on these patients’ outcomes.

## Background

Triage, which stems from the word ‘trier’, meaning to sort, is most known for its use in medical settings.^1,2,3^ The triage process was originally developed during World War I, when the limited resources on the battlefield made it necessary to determine who to prioritise for both care and resources.^2,4^ Today, triage is used to prioritise patients based on their urgency with the goals of ensuring that critically ill patients get timely care and preventing unnecessary mortality.^4^ It is most often seen at points of entry into healthcare facilities, such as emergency centres (ECs). Such a system is particularly useful in times of overcrowding, when EC resources are strained.^4^ A good triage system can distinguish correctly between urgent and non-urgent patients, with reproducible results regardless of who is performing the triage.^4,5,6^ A triage system should ideally have low mis-triage rates. Mis-triage is the extent to which a particular system under- or over-triages a patient relative to their true urgency.^7^ Under-triage occurs when critically ill patients are incorrectly placed in lower urgency categories; over-triage occurs when less ill patients are placed into more urgent categories than is required for their true urgency or illness.^8^ Over-triage rates below 35% and under-triage rates below 5% are considered acceptable in most settings.^8^ Well-known systems, such as the Manchester Triage Scale (MTS), the Canadian triage and acuity scale (CTAS) and the Emergency Severity Index (ESI), were developed in high-income countries. The South African Triage Scale (SATS) is one of the few triage systems purposed-designed for use in low- and middle-income countries (LMICs).^4,9,10,11^

In South Africa (SA), the SATS is used to triage patients.^3^ It has five categories to help providers prioritise time and resources. From the most urgent to the least, they are as follows: red, orange, yellow, green, and blue (deceased).^3^ Triage is commonly performed by a nurse in SA; however, this is, anecdotally, usually a less-experienced junior nurse.^3,12^ A study conducted in rural SA (2018) showed that the SATS is easy to use and that the reliability is not affected when it is used by an untrained healthcare provider.^12^ The SATS has been shown to have under-triage rates of around 9% in more urgent categories (red and orange) and overall over-triage rates of 49%.^12,13^ A study in Kenya confirmed similar rates, finding an under-triage rate of 7% and over-triage rate of 60%.^14^ These rates are above acceptable mis-triage rates as set out by international standards.^8^ Under-triage rates can have deleterious effects for patients in rural settings where resources are few and waiting times longer, but it is unknown how this compares to more urban areas or ECs in private hospitals.^12^ In a district hospital in rural SA in the Eastern Cape, the distribution of patients per category was shown to be the following: red, 2%; orange, 15%; yellow, 37%; and green, 47%.^12^ It is not understood if this distribution is true of all ECs using the SATS or if it is dependent on patient population, and therefore differs in private healthcare settings.

The chosen study hospital is a frequently overcrowded Level 2 Trauma private hospital in Pretoria.^15^ It is not clear if patients are being seen timeously or if the SATS is performed in accordance with the standards for this given population. Prior to this study, the SATS had not been evaluated in this setting. It is also not known what the most common presenting pathology is and whether the available resources are appropriate for treating this.

To address these gaps in knowledge, this study aimed to describe the demographics of this patient population, their triage allocations, resources used, time spent in the EC, and the disposition for each triage category for the year 2018.

## Methods

### Design and setting

A retrospective descriptive study was undertaken in the EC of a Level 2 Trauma accredited private hospital in Pretoria. This hospital has over 300 beds as well as multiple intensive care units (ICUs). It has a 24-h EC and percutaneous coronary intervention unit (PCI). The EC has 20 beds with four resuscitation bays. The unit is typically staffed with 12–13 nurses and 1–3 doctors depending on the time of day. There is only one doctor on the floor between 00:00 and 07:00, and this doctor is also responsible for any emergencies in the wards. The EC sees around 100–120 patients per day, and this includes patients with both medical and trauma-related conditions. There is a radiology department right next to the EC and mobile X-rays can be done within the unit for critically ill or injured patients. There is also an EC phlebotomist who conducts all blood tests and present results for the doctor when ready. If a patient is triaged red, blood tests can be done more urgently, taking around 30 min.

Patients are either triaged as they walk in or at the bedside, if brought in by ambulance. Patients are triaged by a nurse using SATS, in accordance with the guidelines set out by the Emergency medicine society of South Africa (EMSSA). Vital signs of the patient are taken and scored using the triage early warning score (TEWS), which is based on the patient’s age. This together with any discriminators (age-appropriate) are entered onto the online system to generate a triage category. The patient then gets seen according to this category. If a patient is triaged as red, the doctor is immediately informed. Once the patient is discharged from the EC, all clinical information is electronically captured by nursing staff onto the hospital’s online system for record-keeping. This also includes information about what types of resources were used such as blood tests, other laboratory investigations, and radiological investigations.

Data relating to demographics, triage, resources used in EC, and hospital disposition were collected on all patients presenting to the EC during the 2018 calendar year. A 1-year period was chosen to cover all potential seasonal variation.

### Data collection and analysis

All data were originally collected at the time of patient presentation to the EC and stored in an online medical records system. This is done daily as part of the hospital’s own record-keeping and was done prior to this study commencing. The data used for this study was drawn from this system once the approval from the hospital was granted. No clinical records were assessed during this study. The data required for this study were extracted by a gatekeeper chosen by the hospital (SM). Data were then checked and anonymised by a hospital data manager (GR) and entered in Microsoft Excel before being given to the research team for analysis. As the data was descriptive and collected directly from the hospital records, no validated tool was used to collect data.

To meet the aim of the study, only the data relating to demographics (age and sex), application of triage (triage category, presenting complaint, and resources used), times and disposition of patient (discharge or admission) were collected. All patient data were considered for inclusion. The patients with missing data (no triage category or no triage time) and those who were given a random, non-SATS triage category (‘silver’ or ‘follow-up’) were excluded from analysis. As it was not known on what basis these random categories were allocated, these patients were excluded altogether. When looking at the objective of time to be seen by a physician, patients who were triaged but left before being seen by an EC doctor were excluded from this calculation. A lot of inaccurate data capture was found when looking at the objectives addressing time. To address this, if less than 5% of patient data points were missing/incorrect for a specific calcluation, then only those patients were excluded, as this was felt to be small enough not to introduce bias into the results. If it was more than 5%, that calculation was not done. Similar presenting complaints were grouped together where appropriate by one of the researchers (a physician). To reduce bias, if there was any uncertainty, they were left as separate. In terms of resources used, these were divided into laboratory (blood test[s]), electrocardiogram (ECG), urine test, radiology (X-rays, scans, ultrasounds). Each of these categories counted as one resource. The information on resources was taken directly from what was captured on the hospital system and was not based on any formal definition. Descriptive analyses were performed using central tendencies such as means. The data were analysed using Microsoft Excel.

### Ethical considerations

Ethical approval was obtained from the Human Research Ethics Committee of the University of Cape Town. Patient consent is given to the hospital to use their data anonymously on arrival in EC. Consent to study these data was obtained from the hospital manager and the hospital Research Committee. A non-disclosure agreement was signed between the researchers and the hospital to ensure data protection.

## Results

### Patient demographics

A total of 32 328 patients were seen at this hospital’s EC during the year 2018. Of these, 3272 (10%) were excluded either because of a non-SATS triage category being assigned (‘silver’ or ‘follow-up) or missing data. This left 29 055 patients eligible for inclusion (Table 1). A mean 2421 ± standard deviation (8.3%) patients were seen each month. This proportion was generally stable, with the highest number of patients seen in March (*n* = 2759; 9.5%) and the lowest number of patients seen in November (*n* = 2123; 7.3%).

**TABLE 1:** Patient demographics of a private emergency centre in Pretoria for the year 2018.

Variable	*n*	%
**Gender**		
Male	14 409	49.6
Female	14 646	50.4
**Age Groups (years)**		
Paediatrics (0–18)	7985	27.5
< 1	2014	25.2
1–12	4077	51.1
12–18	1894	23.7
Adults (19–60)	16 723	57.6
19–40	10 385	62.1
41–60	6338	37.9
Elderly (> 60)	4347	14.9
61–79	3194	73.5
> 80	1153	26.5

### Triage categories

The most frequently allocated triage category was yellow (*n* = 21 351; 73.5%). No patients were triaged as blue (Table 2). The age group of greater than 60 years was most frequently classified as high acuity (red or orange) (*n* = 1429; 32.9%), followed by age group 19–60 years (*n* = 2562; 15.3%) and age group less than 18 years (*n* = 1052; 13.2%).

**TABLE 2:** Triage category allocations for age, disposition and presenting complaints in a private emergency centre in Pretoria in 2018.

Breakdown of age group, diposition and presenting complaint per triage category	Green		Yellow		Orange		Red	
	*n*	%	*n*	%	*n*	%	*n*	%
**Total patients**	2661	9.1	21 351	73.5	4519	15.6	524	1.8
**Most common age group**								
Adults	-	59.6	-	59.9	-	51.8	-	-
Elderly	-	-	-	-	-	-	-	45.6
**Most common disposition**								
Home	-	89.0	-	81.7	-	49.3	-	-
ICU/High care	-	-	-	-	-	-	-	54.4
**Top presenting complaints**								
Respiratory	-	15.3	-	-	-	-	-	-
Fall	-	-	-	12.1	-	-	-	-
Chest pain	-	-	-	-	-	21.7	-	-
Neurological	-	-	-	-	-	-	-	24.0

ICU, intensive care unit.

Note: Blue category not included as no patients were triaged into this category.

### Presenting complaints

The most common presenting complaint overall in the EC was abdominal pain (*n* = 2613; 8.9%) (Table 3). The most common paediatric complaint was fever (*n* = 1277; 16%), adults was abdominal pain (*n* = 1778; 10.6%) and the elderly was pain not-further-specified (NFS) (*n* = 479; 11.0%). Trauma-related complaints accounted for 12 909 (44.4%) of all presentations.

**TABLE 3:** Top 10 presenting complaints in a private emergency centre in Pretoria (South Africa) in 2018.

No.	Overall PC	*n*	%	Paediatrics PC	*n*	%	Adults PC	*n*	%	Elderly PC	*n*	%
**1**	Abdominal Pain	2613	8.9	Fever	1277	16.0	Abdominal pain	1778	10.6	Pain NFS	479	11.0
**2**	N/V/D	2495	8.5	N/V/D	919	11.5	Pain NFS	1569	9.4	Respiratory	422	9.7
**3**	Pain NFS	2459	8.4	Respiratory	827	10.4	MBA/MVC	1285	7.7	Abdominal pain	314	7.2
**4**	Respiratory	2088	7.2	Sport injury	523	6.5	N/V/D	963	5.8	Fall same level	307	7.1
**5**	MBA/MVC	1516	5.2	Abdominal pain	510	6.4	Chest pain	745	4.5	N/V/D	292	6.7
**6**	Fever	1486	5.1	Fall NFS	476	6.0	Headache	646	3.9	Chest pain	231	5.3
**7**	Fall NFS	1086	3.7	Pain NFS	412	5.2	Back pain	554	3.3	Fall NFS	211	4.9
**8**	Fall same level	924	3.2	Fall same level	219	2.7	Crush injury NFS	538	3.2	Malaise	133	3.1
**9**	Headache	879	3.0	Follow up	162	2.0	Fall NFS	494	3.0	Dizziness	131	3.0
**10**	Sport injury	846	2.9	Headache	158	1.9	Fall same level	398	2.4	Back pain	130	2.9

**Total**		29 055	-		7985	-		16 723	-		4347	-

PC, presenting complaint; N/V/D, nausea/vomiting/diarrhoea; NFS, not further specified; MBA/MVC, motor bike accident/motor vehicle collision.

### Resources used

There were four types of resources used. High acuity patients used the greatest number of resources. Most low acuity patients used no resources at all (Table 4).

**TABLE 4:** Number of patients using zero resources per triage category and number of patients using three or more resources per triage category in a private emergency centre in Pretoria in 2018.

Triage category	Zero resources used		3+ resources used	
	No. of patients	%	No. of patients	%
Red	196 of 524	34.70	92 of 524	17.60
Orange	2488 of 4519	55.10	355 of 4519	7.90
Yellow	15 098 of 21 351	70.70	5 of 21 351	0.02
Green	1921 of 2661	72.20	0 of 2661	0.00

Note: No patients were triaged blue.

### Time to be seen in the emergency centres

**FIGURE 1:**
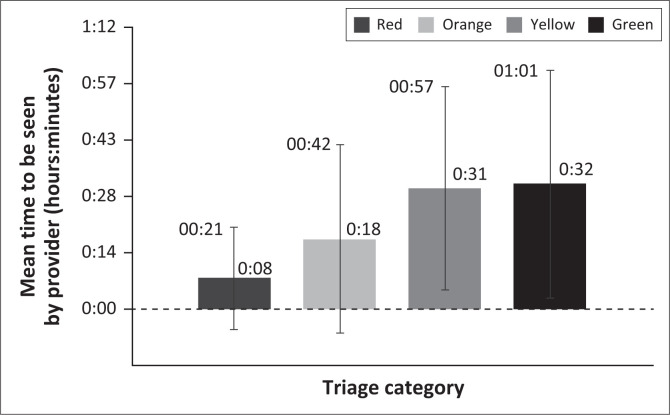
Mean times (with standard deviation) for patients to be seen by emergency centres doctor per triage category in a private emergency centre in South Africa in 2018.

A total of 603 (2.1% of total) patients were excluded from this calculation as they were triaged but not seen by a practitioner (total 28 452 patients). The mean time to be seen by a doctor in the EC from the time of presentation was 28 min. The mean time to be seen by a doctor per triage category has been illustrated in Figure 1.

### Time spent in the emergency centres

A further 163 (0.6%) patients were excluded because of incorrect capture of time, making the total number of patients for this calculation 28 289 (total excluded 766 [2.6%]). Of the 28 289 patients included in this analysis, the mean time spent in the EC was 2 h 20 min and the mean time spent after treatment was 1 h and 54 min (Table 5).

**TABLE 5:** Mean times spent in the emergency centre per triage category (with standard deviations) in a private hospital in South Africa in 2018.

Triage category	Time spent in the EC		Time to be seen by doctor		Time after consult	
	Mean	s.d.	Mean	s.d.	Mean	s.d.
Green	1 h 45 min	1 h 42 min	32 min	29 min	1 h 16 min	1 h 58 min
Yellow	2 h 18 min	2 h 01 min	31 min	26 min	1 h 49 min	1 h 59 min
Orange	2 h 47 min	1 h 47 min	18 min	24 min	2 h 30 min	1 h 46 min
Red	2 h 51 min	1 h 52 min	8 min	13 min	2 h 43 min	1 h 51 min

**Overall mean**	**2 h 20 min**	**1 h 58 min**	**28 min**	**26 min**	**1 h 54 min**	**1 h 57 min**

EC, emergency centre; s.d., standard deviation.

### Area of disposition

Approximately three-quarters (*n* = 22 113; 76.1%) of patients seen in the EC were discharged home. A small proportion were triaged and cancelled their files before being seen, absconded or they were seen and refused hospital treatment (*n* = 682; 2.3%). Of the 38 (0.1%) patients who died in the EC, five were found to be classified as dead on arrival under presenting complaint, but allocated the triage category of red. Of those admitted to the study hospital, 4187 (71.9%) were admitted to a general ward and 1637 (28.1%) were admitted to high care or ICU (Table 6).

**TABLE 6:** Distribution of disposition per triage category in a private emergency centre in Pretoria in 2018.

Area of disposition	Red		Orange		Yellow		Green		Total	
	*n*	%	*n*	%	*n*	%	*n*	%	*n*	%
ICU/HC	285	54.4	1045	23.1	302	1.4	5	0.2	1637	5.6
Ward	108	20.6	995	22.0	2957	13.8	127	4.8	4187	14.4
Transferred	37	7.1	193	4.2	197	0.9	9	0.3	436	1.5
Discharged	58	11.1	2230	49.3	17 440	81.7	2385	89.6	22 113	76.1
Other	36	6.9	56	8.2	455	66.7	135	19.8	682	2.4

**Overall**	**524**	**-**	**4519**	**-**	**21 351**	**-**	**2661**	**-**	**29 055**	**-**

Note: Other = died/refused hospital treatment/assessed and not seen/absconded. No blue patients were assigned from this population. Values are rounded and so may not equate to 100.

ICU, intensive care unit; HC, high care.

## Discussion

This study gives insight into the demographics, distributions of triage categories, and triage outcomes within this private hospital’s EC. The majority of patients presenting to the EC were considered by the SATS to be low acuity (yellow and green patients), and a small proportion were categorised as high acuity (red and orange). The distribution of high and low acuity patients was comparable to that seen in a 2018 study in a rural district hospital in SA^12^; however, there were differences in specific categories. This study’s private EC saw far more yellow patients (73.5% in this study vs. 37%), whereas the rural hospital saw far more green patients (46% vs. 9.2% in this study).^12^ The reason for this discrepancy could be as a result of this study’s population having access to general practitioners in the community. These patients may seek care from those providers and thus, not use the EC for non-urgent illnesses. In rural SA, the EC might be the closest or most available point of care for certain patients, even if inappropriate. It is also not known whether a senior healthcare worker was involved in triage at this study’s EC, and whether this impacted the numbers of patients assigned per category.

The finding of no patients triaged as blue (deceased) on arrival was also in keeping with the study in rural SA.^12^ However, five of the patients who died in the EC were classified under the presenting complaint as ‘dead on arrival’, but triaged red. If these patients were, indeed, deceased upon arrival, they should have been triaged blue by SATS definition, which would then change the results of this study. As this study did not look at clinical notes or patient vitals, it is unclear as to why this was the case. Further research is needed to determine whether these patients were incorrectly triaged or whether there were in fact signs of life on arrival. If these patients are found to be incorrectly triaged, then this will highlight a gap in training that can be addressed to improve the accuracy of triage.

Trauma-related complaints formed almost half (44.4%) of the total presenting complaints in this study. This proportion is more than what was found in a study conducted in Paarl, SA (a government hospital), which showed that only 36% of patients presented with trauma-related conditions, although the sample size in this study was considerably smaller.^16^ A reason that this proportion could be higher is because many private institutions, including the one in this study, also treat patients for the Worker’s Compensation Assistance (WCA) fund. These are patients who generally have minor injuries that could be treated at primary care facilities. However, as the WCA fund has designated places for care, this forces more patients to seek treatment at a facility that is inappropriate. South Africa is known to have one of the highest rates of violence and trauma in the world.^16^ This finding in this private hospital study is consequently very much in keeping with SA’s national statistics, where trauma is one of the leading causes of morbidity.^16^

Red and orange categories were found to use the greatest number of resources, which is consistent with more critical diagnoses where a patient may have multiple pathologies. This is in keeping with the ESI findings where higher acuity categories require more resources.^6,17^ Of note was the fact that over 30% of red and orange patients used no resources at all. Whilst high acuity patients are usually expected to be the sickest by definition, they can likewise be triaged as more urgent based on a time-sensitive need. For example, patients with a simple dislocated shoulder get placed into the SATS orange triage category; however, diagnosis can be made clinically. Nevertheless, given that litigation is common in private healthcare, one would expect all high acuity patients to use at least one resource to avoid missing a critical diagnosis. In the dislocated shoulder example, the minimum would be to use X-rays to confirm a successful reduction. Therefore, this finding of using no resources for some critical patients appears illogical in a setting where litigation is high. Another possible explanation for this finding is that if a patient is so critical, the focus is on providing life-saving treatment. Notes are sometimes then written retrospectively, and resources may accidently be left out of clinical notes, resulting in inaccurate data capture. Further research is required to see if these findings are because of a clinical reason or inadequate data capture.

High acuity patients (red and orange) in this study were found to wait to be seen by a doctor outside the recommended standards set by SATS. The orange and red patients at Zithulele Rural Hospital in the Eastern Cape were also seen after the recommended time frames.^12^ The difference in time to see red patients between studies was on average only 3 min (11 min vs. 8 min).^12^ However, no confidence intervals were available to determine if these findings are statistically significant. As these are usually the sickest patients, this raises concerns. This is because triage aims to identify critically ill patients early in order to provide rapid treatment to reduce morbidity and mortality. Whilst hospitals in rural SA may have good reason to explain these delays, such as fewer available staff, it is unclear why this was also the case in a better-resourced EC. This is especially concerning because, typically, the doctor is given the orange or red file in their hand immediately upon the patient’s arrival. One major contributing factor could be that, when the unit is busy, there may be access block to beds for patients. Therefore, even if the doctor is ready, the patient may not yet be in a bed, increasing the time to be seen. Another possible reason for these delays could be because of the way in which the EC is designed. The different sections can be far from each other and so the doctor is not aware of a new red patient if they are otherwise occupied. Until such a patient is brought to a doctor’s attention, there will be delays in them receiving treatment. Whilst re-building the EC is not necessarily practical, a system such as the ringing of a bell throughout the EC to indicate a red arrival could be implemented. This would alert the doctor immediately and possibly improve the delivery of care. The exact reasons for major delays at this EC are not known and this warrants further exploration, as these delays may have adverse outcomes for patients and go against the purpose of triage. This study did not follow patients to understand if these delays affected outcomes, a topic that warrants further analyses.

The mean time spent in the EC was greater than 2 h, and the higher the acuity of the patient, the more time was spent. This finding is in keeping with the study by Hocker et al. (2011) which showed that, when using the ESI, higher acuity patients also spent longer in the EC.^6,17^ It was suggested that more resources and investigations were used for these patients, but whether that was the main reason for the prolonged stay is not clear.^6,17^ One possible explanation is that a polytrauma patient may require reduction and casting of fractures and a head-to-pelvis scan to determine the extent of the injuries. The scans are done in another department and, alongside procedure times, can increase patient stays in the EC. However, for less complicated critically ill patients, this still seems to be too long. In this EC, if a patient presents with an ST-segment elevation myocardial infarction (STEMI), they can be in the percutaneous catherisation intervention (PCI) unit within 15 min – 30 min. Furthermore, for all red patients, bloods can be run urgently, with results completed 30 min faster than for non-urgent patients. This implies that the time to final diagnosis should be sooner. These long EC wait times are cause for concern because they result in definitive treatment being delayed. Definitive treatment will naturally vary from patient to patient. For some, it may mean theatre or ICU care; for others, it may be simply antibiotics. If this EC is full, the staff-to-patient ratio is skewed, meaning that items are more likely to be missed or forgotten. This can impact on general patient care for both patients requiring admission and new patients presenting to the EC. It also potentially increases the chance for a major adverse outcome in critically ill patients. Patients who require more one-on-one care, such as ventilated patients, could easily deteriorate unnoticed in a chaotic environment. The reasons for these delays are not clearly understood and would need to be investigated to determine if they can be reduced.

In terms of disposition, this study found that the majority of patients were discharged home; the discharge rate was 20% higher than that of Meyer et al.’s (2018) study in rural SA.^12^ One explanation for this may be that patients in this study have better access to care and can follow up more easily and are therefore discharged more easily. However, there is also a possibility that the burden of disease may differ greatly and that could explain the difference in findings. Final diagnoses of this study’s population may provide a clearer idea of why patients were admitted or discharged. Furthermore, if the majority of patients are yellow and being discharged, this raises the question of whether the current EC is optimally set up for the patients it sees. With these results, it may be worth considering having a larger area for yellows with more dedicated staff, so that these patients can be fast-tracked, thus preventing access block or drawing away of resources from more critical patients.

Most red patients in this study were admitted, with the majority being admitted to high care or ICU. These findings are in line with international studies done on the ESI and MTS which showed that high acuity patients were more likely to be admitted.^18,19^ The ESI showed that 48% of red category patients were admitted to ICU, whilst the rest were admitted to the wards.^8,19^ This study in a private hospital in SA showed high numbers of orange patients discharged home with equal numbers admitted to the ward or high care/ICU. Some red patients in this study were also discharged home. This is in contrast to the ESI which saw no red patients discharged and only 22% of orange patients discharged.^8,19^ In Meyer et al.’s 2018 study in rural SA, high discharge rates for red and orange patients were also noted, but with higher admission rates for green patients (9.4% vs. 5%).^12^ Another study which was conducted by assessing the use of SATS in various low-income countries by Dalwai et al., showed that the majority of red patients seen were also discharged (exceeding 50%).^20^ The fact that so many high acuity patients were discharged when using the SATS may imply that either triage is being incorrectly performed or the system itself tends to over-triage. It is not known whether a senior healthcare worker was used in this study to determine the final triage category, as set out by the SATS. Given that many studies using the SATS in a variety of settings are showing consistent results, it would suggest the latter as a reason for these findings. This would need to be investigated to determine the real implications of these findings, however.

### Limitations of study

This study had several limitations. The data were captured online by different people. This meant that some data had to be excluded because of inaccurately recorded times (some were captured using 24-h clock, others not), reducing this study’s sample size and potentially skewing the results. Although this affected the calculations regarding time the most, inaccurate data capture could have affected all variables in some manner. Next, was the allocation of the silver category to some patients. This is not a formal SATS category. It is not clear on what criteria this is based, who made that decision, or at what point in the process this was decided (i.e. at triage or when captured online by hospital staff). As it is possible that these patients could have been categorised into a formal SATS category, excluding these patients may have impacted overall numbers per triage category. Furthermore, presenting complaints were grouped together by the researcher who is a physician (e.g. abdominal pain and gastrointestinal [GIT]-abdominal pain). Although any ambiguities were left in original categories to reduce bias, this was still a subjective process and so may have impacted the results. This study was conducted only at one hospital and thus may not hold external validity as it is not representative of all the private institutions. Repeating this study across multiple facilities to see whether the results are reproducible would be of use for those considering SATS implementation in similar settings.

## Conclusion

This study shows that most patients attending this private hospital EC fell into the age category of 19–60 years. Most patients were triaged into low acuity categories, which is in keeping with the high discharge rate seen. This does not appear to be unique to a private healthcare setting, although the reasons for discharge may be different because of better access to healthcare and the ability to follow up more easily. Even though most patients are being seen in a timely manner, high acuity patients are waiting slightly longer than the recommended times. This is unexpected in a private healthcare setting, where resources are more readily available. The reasons for these delays are not apparent and requires further exploration as this issue can impact patient outcomes substantially. High acuity patients were also found to spend the longest amount of time in the EC, after being seen by the doctor, but reasons for this remain unclear. Additionally, high acuity patients were shown to use the greatest number of resources and to spend the longest time in the EC. There may be a correlation between these two results, but further research is needed to confirm and correct this. A fair proportion of high acuity patients was also noted to use no resources which was unexpected as these patients are usually the sickest. With more readily available resources in private healthcare as well as high risk of litigation, one would have presumed that all high acuity patients would have had an investigation of some kind. Moreover, whilst most high acuity patients are admitted to high care or ICU, a great number were also discharged home. This raises concerns about the accuracy of triage in this private facility.
